# Multicenter comparative study of *Enterocytozoon bieneusi* DNA extraction methods from stool samples, and mechanical pretreatment protocols evaluation

**DOI:** 10.1038/s41598-024-66154-2

**Published:** 2024-07-04

**Authors:** Céline Nourrisson, Maxime Moniot, Maxime Tressol, Céline Lambert, Emilie Fréalle, Florence Robert-Gangneux, Damien Costa, Louise Basmaciyan, Philippe Poirier

**Affiliations:** 1 Parasitology-Mycology Department, CHU Clermont-Ferrand, 3IHP, 58 Rue Montalembert, 63000 Clermont-Ferrand, France; 2 Microbes, Intestin, Inflammation et Susceptibilité de l’Hôte, M2iSH, UMR INSERM 1071, Clermont Auvergne University, Clermont-Ferrand, France; 3 National Reference Center (NRC) for Cyptosporidiosis, Microsporidia and Other Digestive Protozoa, Clermont-Ferrand, France; 4 Biostatistics Unit, DRCI, CHU Clermont-Ferrand, Clermont-Ferrand, France; 5 Laboratory of Parasitology-Mycology, CHU Lille, 59000 Lille, France; 6 CHU Rennes, Inserm, EHESP, Irset, UMR_S 1085, Univ Rennes, Rennes, France; 7 Laboratory of Parasitology-Mycology, EA7510 ESCAPE, University Hospital of Rouen, Univ Rouen Normandie, Normandy, France; 8 Parasitology-Mycology Department, CHU Dijon, Dijon, France; 9 National Reference Center (NRC) for Cyptosporidiosis, Microsporidia and Other Digestive Protozoa, Rouen, France; 10 National Reference Center (NRC) for Cyptosporidiosis, Microsporidia and Other Digestive Protozoa, Dijon, France

**Keywords:** Gastroenterology, Microbiology, Parasitology, Molecular medicine

## Abstract

Nowadays, the use of qPCR for the diagnosis of intestinal microsporidiosis is increasing. There are several studies on the evaluation of qPCR performance but very few focus on the stool pretreatment step before DNA extraction, which is nevertheless a crucial step. This study focuses on the mechanical pretreatment of stools for *Enterocytozoon bieneusi* spores DNA extraction. Firstly, a multicenter comparative study was conducted evaluating seven extraction methods (manual or automated) including various mechanical pretreatment. Secondly, several durations and grinding speeds and types of beads were tested in order to optimize mechanical pretreatment. Extraction methods of the various centers had widely-varying performances especially for samples with low microsporidia loads. Nuclisens® easyMAG (BioMérieux) and Quick DNA Fecal/Soil Microbe Microprep kit (ZymoResearch) presented the best performances (highest frequencies of detection of low spore concentrations and lowest Ct values). Optimal performances of mechanical pretreatment were obtained by applying a speed of 30 Hz during 60 s with the TissueLyser II (Qiagen) using commercial beads of various materials and sizes (from ZymoResearch or MP Biomedicals). Overall, the optimal DNA extraction method for *E. bieneusi* spores contained in stool samples was obtained with a strong but short bead beating using small-sized beads from various materials.

## Introduction

Molecular biology approaches for the diagnosis of digestive parasitoses are booming, due to (i) the higher supply of commercial kits, some of which target various pathogens (multiplex kits)^1–4^, and to (ii) the difficulty of maintaining staff skills for microscopy. Although this is an essential step, DNA extraction methods are not standardized at this time, which influences the reliability of the results of the amplification stage. Importantly, stools are complex biological matrices where parasites can be present in the form of a fragile and easy-to-lyse structure (*i.e.* vegetative stage, larvae…), but also in a much more solid and resistant wall (*i.e.* ova, cyst, oocyst, spore… according to the parasite). For these so-called resistant forms, in addition to the enzymatic lysis, conventionally used for DNA extraction, mechanical and/or chemical pretreatment steps will be crucial to destroy these structures and optimize the extraction yield. As an example, a mechanical grinding step using beads and a high-frequency oscillating stirrer have been proposed as a suitable solution in order to optimize the DNA extraction from *Cryptosporidium* oocysts^5,6^.

Intestinal microsporidiosis manifests as profuse watery diarrhea and can affect immunocompromised but also immunocompetent subjects^7^. *Enterocytozoon bieneusi* is the main species responsible for this disease in humans. Spores are the infectious stage of microsporidia and are characterized by their thick and difficult-to-break chitin wall, giving them a great environmental resistance. Their size varies according to the species, and reaches approximatively 1.5 × 0.9 µm for *E. bieneusi*^7^. Diagnosis can be based on microscopic examination of a stool smear after appropriate staining (chromotrope-based staining or chemifluorescent optical brightening agents) or by immunofluorescence. However this approach may lack sensitivity and needs experienced microscopists. Molecular diagnosis solves these pitfalls^8^. Whereas the limit of detection with classical staining or fluorescent stains that bind chitin in the spore wall is 50,000 organisms/mL, PCR-based methods have been shown to be able to detect 100 to 1000 spores/mL in clinical samples^7–9^.

In this context, the aim of this study was to provide data on the benefits of stool mechanical pretreatment for the diagnosis of intestinal microsporidiosis. This study consisted of two parts: (i) seven *E. bieneusi* DNA extraction methods including mechanical pretreatment step were compared in a multicenter study, and (ii) several parameters of mechanical pretreatment step (speed and duration of grinding, and size and type of beads) were tested in order to optimize the grinding protocol.

## Results

### Part 1: Multicenter comparative study of seven *E. bieneusi* DNA extraction methods

In total, seven methods, using four different grinders (oscillating movement and vortex homogenizer), five types of beads, five lysis buffers and five grinding programs, were compared (Table 1).

**Table 1:** Main characteristics of each DNA extraction method including mechanical pretreatment protocol.

	Center 1		Center 2	Center 3	Center 4		Center 5
	Method 1	Method 2	Method 3	Method 4	Method 5	Method 6	Method 7
Stool test sample (µL)	200		400	150	250		200
Beads	0.5 mm glass beads (Next Advance)		1.4 mm ceramic + 0.112 mm silica + one 4 mm glass beads (MP Biomedicals)	0.1 and 0.5 mm ZR BashingBead® (ZymoResearch)	0.7 mm garnet beads		1.4 mm ceramic beads
Grinder	TissueLyser® II (Qiagen)		FastPrep 24 (MP Biomedicals)	MagnaLyser® (Roche Diagnostics)	Vortex-Genie 2® (Scientific industries)		MagnaLyser® (Roche Diagnostics)
Grinding protocol	3 min – 30 Hz		1 min – 6 m/s	45 s – 7000 rpm	10 min – 3200 rpm		1 min – 3500 rpm
DNA extraction kit or lysis buffer	Nuclisens® easyMAG lysis buffer (BioMérieux)	DNA Stool Minikit (Qiagen)	Nuclisens® easyMAG lysis buffer (BioMérieux)	BashingBead® Buffer, Quick DNA Fecal/Soil Microbe Microprep kit (ZymoResearch)	PowerBead Solution QIAamp PowerFecal DNA kit (Qiagen)	DNA tissue kit (Qiagen)	Bacterial lysis buffer (Roche Diagnostics)
DNA extraction apparatus	Ingenius (Elitech)	None (manual extraction)	None (manual extraction)	None (manual extraction)	None (manual extraction)	EZ1 Advanced XL (Qiagen)	MagnaPure 96 System® (Roche Diagnostics)

No PCR inhibition was noted. All negative controls (0 spore/mL) included in the study were negative. Details of PCR Ct values obtained for each method are reported in Table S2.

**Figure 1:**
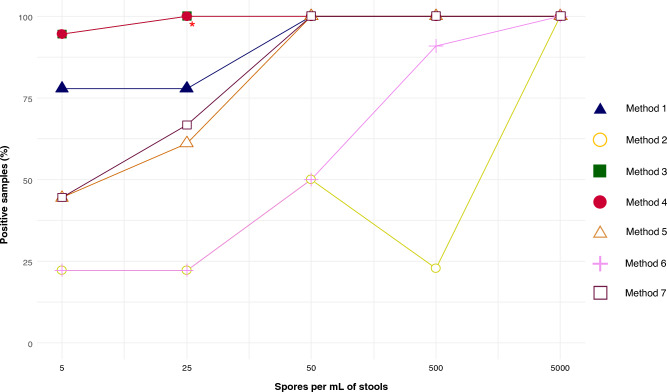
Percentage of positive *E. bieneusi* qPCR for each method according to spore concentration. * The sample sizes were n = 18 at the concentrations 5, 25 and 50 spores/mL, and n = 22 at the concentrations 50, 500 and 5,000 spores/mL, except for method 4 at a concentration of 25 spores/mL for which two replicates were not performed (not enough DNA).

From a qualitative point of view (*i.e.* positive or negative result, Fig. 1, Table 2), there was no difference between the methods for the highest concentration (5,000 spores/mL), as all tested conditions and replicates were positive. For the concentrations of 500 and 50 spores/mL, method 2 and method 6 (to a lower extent) yielded poor performances with 22.7% and 90.9% positive PCRs, and 50% and 50% positive PCR, for 500 spores/mL and 50 spores/mL, respectively. The remaining methods gave positive results for all replicates at these two concentrations. For the two lowest concentrations (5 and 25 spores/mL), the differences between methods were more pronounced. Methods 5 and 7 had comparable detection rates, which decreased in parallel with the spore concentration. Method 1 had a detection rate of 77.8% for both concentrations. The two methods allowing a significantly improved detection were methods 3 and 4, each yielding a single negative result at the concentration of 5 spores/mL, *i.e.* a detection rate of 94.4%. Method 3 reached 100% detection for 25 spores/mL, but we could not compare to method 4 as a technical problem (not enough DNA) did not allow the center to carry out all replicates. Apart from this problem, these two methods would have presented the same analytical performance.

**Table 2:** Comparison of the percentage of positive *E. bieneusi* qPCR according to the pretreatment/extraction methods and spore concentration.

	n (%)	*p*^1^	*p*^2^	*p*^3^	*p*^4^	*p*^5^	*p*^6^
5 spores per mL							
Method 1	14/18 (77.8)						
Method 2	4/18 (22.2)	0.01					
Method 3	17/18 (94.4)	0.90	< 0.001				
Method 4	17/18 (94.4)	0.90	< 0.001	1.00			
Method 5	8/18 (44.4)	0.57	0.91	0.02	0.02		
Method 6	4/18 (22.2)	0.01	1.00	< 0.001	< 0.001	0.91	
Method 7	8/18 (44.4)	0.57	0.91	0.02	0.02	1.00	0.91
25 spores per mL							
Method 1	14/18 (77.8)						
Method 2	4/18 (22.2)	0.01					
Method 3	18/18 (100)	0.53	< 0.001				
Method 4	16/16* (100)	0.53	< 0.001	1.00			
Method 5	11/18 (61.1)	0.98	0.36	0.08	0.08		
Method 6	4/18 (22.2)	0.01	1.00	< 0.001	< 0.001	0.36	
Method 7	12/18 (66.7)	1.00	0.17	0.17	0.17	1.00	0.17
50 spores per mL							
Method 1	18/18 (100)						
Method 2	9/18 (50.0)	0.006					
Method 3	18/18 (100)	1.00	0.006				
Method 4	18/18 (100)	1.00	0.006	1.00			
Method 5	18/18 (100)	1.00	0.006	1.00	1.00		
Method 6	9/18 (50.0)	0.006	1.00	0.006	0.006	0.006	
Method 7	18/18 (100)	1.00	0.006	1.00	1.00	1.00	0.006
500 spores per mL							
Method 1	22/22 (100)						
Method 2	5/22 (22.7)	< 0.001					
Method 3	22/22 (100)	1.00	< 0.001				
Method 4	22/22 (100)	1.00	< 0.001	1.00			
Method 5	22/22 (100)	1.00	< 0.001	1.00	1.00		
Method 6	20/22 (90.9)	0.90	< 0.001	0.90	0.90	0.90	
Method 7	22/22 (100)	1.00	< 0.001	1.00	1.00	1.00	0.90

* Two replicates were not performed (not enough DNA).

*P*-values are presented as *p*^1^: Comparison with method 1, *p*^2^: Comparison with method 2, etc. (Chi-squared test).

**Figure 2:**
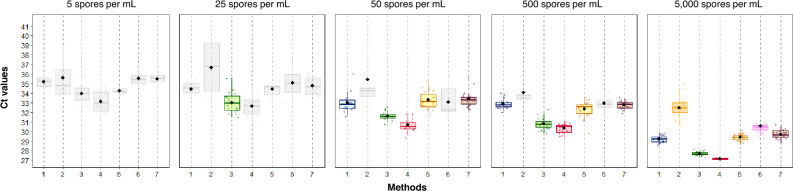
Ct values of *E. bieneusi* qPCR for each method according to spore concentration. Light grey boxplots correspond to partial detection or incomplete analysis of replicates (*i.e.* method 4 at 25 spores/mL). Boxplots show median and 25th and 75th percentiles, the upper whisker is the largest value no further than 1.5 interquartile range from the hinge, and the lower whisker is the smallest value at most 1.5 interquartile range of the hinge.

From a quantitative point of view (*i.e.* Ct values, Fig. 2), statistical analyses were performed only when all replicates from a condition were positive (Table 3). Methods 2 and 6 had significantly higher mean Ct values for the concentration of 5,000 spores/mL (32.48 ± 1.00 and 30.55 ± 1.11, respectively), whereas methods 3 and 4 had significantly lower mean Ct values (27.66 ± 0.20 and 26.80 ± 0.27, respectively). Methods 1, 5 and 7 had intermediate mean Ct values. As expected, as the concentration of spores decreased, the average of Ct values increased for each method, while keeping the same ranking in terms of detection. Between 50 and 500 spores/mL, the average Ct values only decreased from 0.1 Ct (method 1) to 1 Ct (method 5), but the dispersion of the values increased, for example ΔCt = 4.15 for 50 spores/mL and ΔCt = 1.95 for 500 spores/mL with method 1. For the stool suspension at 25 spores/mL, all qPCR replicates were positive only with method 3, but it should be noted, as previously mentioned, that two qPCR replicates could not be run for method 4 because the volume of eluate was insufficient (all the other replicates were positive). For the lowest concentration (5 spores/mL), none of the methods yielded positive qPCR results. Mean Ct values for the two methods with the maximum of positive qPCR replicates, *i.e.* methods 3 and 4, were 33.88 ± 0.47 and 34.04 ± 1.48 and 32.41 ± 0.96 and 33.45 ± 1.47, respectively (Fig. 2).

**Table 3:** Comparison of Ct values of *E. bieneusi* qPCR according to the pretreatment/extraction methods and spore concentration.

	n/N	mean ± SD	*p*^1^	*p*^2^	*p*^3^	*p*^4^	*p*^5^	*p*^6^
50 spores per mL								
Method 1	3/3	33.0 ± 0.9						
Method 2	0/3	nc	nc					
Method 3	3/3	31.6 ± 0.5	< 0.001	nc				
Method 4	3/3	30.7 ± 0.6	< 0.001	nc	0.003			
Method 5	3/3	33.3 ± 0.9	1.00	nc	< 0.001	< 0.001		
Method 6	1/3	nc	nc	nc	nc	nc	nc	
Method 7	3/3	33.4 ± 0.8	1.00	nc	< 0.001	< 0.001	1.00	nc
500 spores per mL								
Method 1	2/2	32.9 ± 0.5						
Method 2	0/2	nc	nc					
Method 3	2/2	30.8 ± 0.6	< 0.001	nc				
Method 4	2/2	30.3 ± 0.5	< 0.001	nc	0.09			
Method 5	2/2	32.3 ± 0.9	0.4	nc	< 0.001	< 0.001		
Method 6	0/2	nc	nc	nc	nc	nc	nc	
Method 7	2/2	32.8 ± 0.4	1.00	nc	< 0.001	< 0.001	0.16	nc
5000 spores per mL								
Method 1	2/2	29.2 ± 0.4						
Method 2	2/2	32.5 ± 1.0	< 0.001					
Method 3	2/2	27.7 ± 0.2	< 0.001	< 0.001				
Method 4	2/2	26.8 ± 0.3	< 0.001	< 0.001	0.02			
Method 5	2/2	29.4 ± 0.3	1.00	< 0.001	< 0.001	< 0.001		
Method 6	2/2	30.6 ± 1.1	< 0.001	< 0.001	< 0.001	< 0.001	< 0.001	
Method 7	2/2	29.7 ± 0.4	1.00	< 0.001	< 0.001	< 0.001	1.00	0.02

*P*-values are presented as *p*^1^: Comparison with method 1, *p*^2^: Comparison with method 2, etc. Comparisons were made only when all replicates from a given condition were positive. n/N: Number of positive eluates /number tested; nc: Not calculated; SD: Standard deviation.

### Part 2: Optimization of parameters of mechanical pretreatment

**Figure 3:**
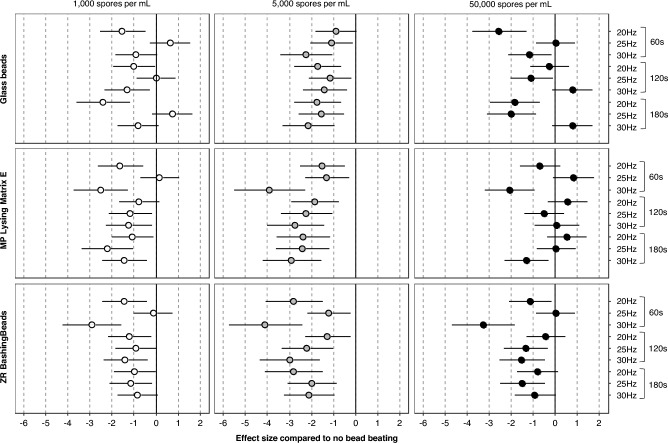
Effect size of the comparison of Ct values between each type of beads and no bead beating according to spore concentration and mechanical pretreatment (combination of frequency and duration). Data are presented as effect size and 95% confidence interval, and interpreted as follows: 0.2 = small effect, 0.5 = medium effect and 0.8 = large effect. A positive effect size means that the Ct values are higher than in the “no bead beating” condition (and vice versa if the effect size is negative). The larger is the effect size (in absolute value), the larger is the difference. If the confidence interval does not intersect “0”, the difference is significant.

The details of Ct values are reported in Table S3. The highest Ct values were essentially obtained in control samples that were not submitted to a grinding step (Fig. 3). Indeed, regardless of the type of beads used, effect sizes between grinding step and no bead beating varied between − 2.92 (95%CI − 4.24 to − 1.57) and 0.73 (95%CI − 0.19 to 1.64) for concentration of 1000 spores/mL, between − 4.11 (95%CI − 5.75 to − 2.43) and − 0.91 (95%CI − 1.83 to 0.04) for 5000 spores/mL, and between − 3.27 (95%CI − 4.68 to − 1.83) and 0.83 (95%CI − 0.11 to 1.74) for 50,000 spores/mL (Fig. 3).

Overall, the Ct gain with bead beating was significant in most conditions (Fig. 3), but clearly associated with spore load. Then, for low (1000 spores per mL) and high (50,000 spores per mL) spore loads, the Ct gain was less important than for medium load (5000 spores per mL).

Depending on the type of bead considered, the optimal grinding protocols (*i.e.* speed, duration) were not the same. For example, considering the concentration of 50,000 spores/mL, the average of Ct values was significantly lower with glass beads at 20 Hz during 60 s than with MP Lysing Matrix E beads or ZR BashingBeads beads (20.15 ± 0.51 and 21.11 ± 0.56, *p* < 0.001 or 20.98 ± 0.37, *p* = 0.003, respectively). For the same concentration, the average of Ct values was significantly higher with glass beads at 30 Hz during 180 s than with MP Lysing Matrix E beads or ZR BashingBeads beads (21.90 ± 0.45 and 20.81 ± 0.50, *p* < 0.001 or 20.95 ± 0.63, *p* < 0.001, respectively).

Depending on the spore concentration considered, the optimal grinding protocols and beads were not the same. For example, considering the protocol 25 Hz during 180 s, the average of Ct values was significantly higher with glass beads at the concentration of 1000 spores/mL than with MP Lysing Matrix E beads (28.24 ± 0.68 and 26.04 ± 0.41, *p* < 0.001). For the same protocol, the average of Ct values was significantly lower with glass beads at the concentration of 50,000 spores/mL than with MP Lysing Matrix E beads (20.44 ± 0.51 and 21.52 ± 0.56, *p* = 0.002).

Overall, considering ZR BashingBeads or MP Lysing Matrix E beads, whatever the spore concentration, the lowest mean Ct values were obtained with a grinding of 30 Hz during 60 s (Table S3). With this protocol, the average of Ct values was significantly lower compared with glass bead protocols: at 50,000 spores/mL when using ZR BashingBeads (20.52 ± 1.02 and 19.25 ± 0.78, *p* = 0.02) or at 5000 spores/mL when using MP Lysing Matrix E beads (23.21 ± 1.39 and 21.94 ± 1.01, *p* = 0.03).

## Discussion

Optimized extraction protocols combined with sensitive and specific PCR assays are necessary to guarantee the detection of low loads of Microsporidia spores in stool specimen. Stool is a complex biological matrix, and it is now widely recognized that a pretreatment of the sample, before DNA extraction, is essential^12–17^. Various studies evaluated qPCR for microsporidia diagnosis, but few of them focused on the DNA extraction step^17,18^.

Whatever the pretreatment method chosen (*i.e.* chemical, enzymatic, mechanical), an evaluation is essential. The present study describes the optimization of a mechanical pretreatment whose principle is based on the dilution of a stool specimen in a lysis buffer containing beads which will induce the grinding of the sample under the effect of high-speed agitation. Optimizing the step of mechanical pretreatment can prevent DNA fragmentation and the release of PCR inhibitors. In a previous study, we showed that the detection of the intestinal parasite *Cryptosporidium* was greatly impacted by the extraction step, and that aggressive pretreatment could result in a decrease in the qPCR performances^5^.

The design of this study took into account several pitfalls that could skew results. Aliquots of stool were kept at 4 °C. Indeed, a previous study tested various storage conditions before *Encephalitozoon intestinalis* DNA extraction and demonstrated that the limit of detection was lower when stools were analyzed fresh or after storage at + 4–8 °C, compared to frozen or kept at room temperature^9^. Furthermore, in order to guarantee DNA integrity, all PCR were performed within 10 days after DNA extraction and extracts were kept at 4 °C, avoiding freezing/thawing cycles. Finally, the number of DNA extractions and of DNA amplifications for each concentration and protocols tested were optimized according to the Poisson’s law, to warrant the robustness of the statistical analyses.

In the comparative study of DNA extractions methods (part 1), allowing the comparison of methods used in five French medical Parasitology laboratories, methods using Nuclisens® easyMAG (BioMérieux) and Quick DNA Fecal/Soil Microbe Microprep kit (ZymoResearch) showed the best performances and were able to detect as low as 5 spores/mL in almost 95% of cases. High performances of Nuclisens® easyMAG were previously reported for *Cryptosporidium* and *Toxoplasma gondii* DNA extraction from stool and amniotic fluid, respectively^6,19^. This system is also known to limit the co-extraction of PCR inhibitors^20^. Furthermore, ZymoResearch fecal extraction kits, which include an optimized mechanical pre-treatment step with beads and lysis buffer provided in the kits, were previously reported to yield excellent performances for *Cryptosporidium* DNA extraction^6,21^. At the opposite, methods using DNA Tissue kit and DNA Stool Mini kit (Qiagen) had the lowest performances and were proven deficient for 50 and 500 spores/mL, respectively. These results are coherent with those of a previous study where QIAamp DNA Mini kit allowed to detect till 100 spores/mL and had a higher ability to extract Microspordia DNA than stool kit^17^.

Due to the huge differences in performances between methods, we focused further on various parameters implied in mechanical pretreatment. Firstly, the physicochemical characteristics (*i.e.* size, shape, materials) of the beads can impact the effectiveness of the pretreatment step. Indeed, size of beads should be of an order of magnitude comparable to that of the parasite to be grinded. For example, small glass beads maximize sporocyst release from *Eimeria* oocysts, compared to larger ones^20^. Depending on their shape (spherical, irregular), the beads will not exert the same type of physical forces^20,21^. Their chemical composition influences their hardness and density. For example, glass beads associated with freezing–thawing have been shown to increase the concentration of DNA extracted from *E. intestinalis*^15^. Secondly, the choice of grinding speed and duration that were tested here was based on a previous study on *Cryptosporidium* oocysts, where the best parameters were a lower speed and an intermediate duration of grinding, *i.e.* 4 m/s and 60 s on the FastPrep 24® (MP Biomedicals) grinder/homogenizer^6^. The potential deleterious effect of excessively long grinding times or excessively high speeds (which can lead to DNA degradation) must also be taken into account.

Our results showed that the implementation of a grinding protocol must take into account the association of the type of beads and the grinding program. Indeed, in the present study, the 0.5 mm glass beads performed better than the other types of beads tested when shook at 20 Hz, particularly for 60 s, while these glass beads are the least efficient at 30 Hz. The choice of the best protocol of grinding and beads type should be deduced from the results obtained on the lowest quantity of spores, *i.e.* the most challenging condition. As expected, the gain on the Ct value was more marked for the lowest concentrations (essentially 5,000 spores/mL, but also 1000 spores/mL). Overall, optimal performances of mechanical pretreatment were obtained by applying a speed of 30 Hz during 60 s using ZymoResearch or MP Biomedicals beads. Of note, the two best methods of the first part of this study were also those using ZR BashingBeads or MP Lysing Matrix E beads, highlighting the major added-value of these beads mixing various bead materials and sizes.

We assume that this work has several limitations. It studies only the impact of oscillations speed and duration, but it should be noted that the type of grinder could also play a major role in the process, as the direction of applied forces is essential (horizontal or vertical oscillation, vortexing). In the same way, choice of lysis buffer/DNA extraction kit is also of great importance, as shown with methods 1 and 2 on the one hand, and 5 and 6 on the other hand, which used the same beads and grinding conditions, but led to very different performances. Various sample diluents were previously tested and it was demonstrated that tissue lysis buffer allowed more efficient DNA extraction than did lysis binding buffer or fetal bovine serum^9^. Finally, only one type of stool (liquid stool) was tested, and it is likely that consistency could also have an impact during grinding.

In conclusion, a short duration of high-speed grinding with a mixture of beads of various materials and of small size is effective for *E. bieneusi* DNA extraction. However, parameters of stool pretreatment and extraction method are linked to one another and need specific optimization work according to the materials available in each laboratory.

## Methods

### Ethical statement

This study was carried out under the supervision of the French National Reference Center for “Cryptosporidiosis, Microsporidia and other digestive protozoa” and benefited from agreements with the French Data Protection Agency (CNIL) and certification by the ethics committee. The samples were sent to the laboratory for a diagnosis of microsporidiosis, and patients were informed that, unless they objected, these samples could be used for research purposes. The use of the samples for scientific purposes was authorized under the number #DC-2022–4982.

### Part 1

Five centers participated to the first part of the study aiming at comparing seven DNA extraction methods (two centers performed two different methods). All extraction protocols included a mechanical pretreatment step.

#### Stool specimen

From a positive stool for *E. bieneusi* (genotype C) whose spore concentration was counted after Calcofluor white staining, dilutions of spores were prepared in a microsporidia-negative liquid stool, to obtain concentrations ranging from 5 to 5000 spores/mL. The coordinating center (university hospital of Clermont-Ferrand, France) transmitted to each participating center, aliquots containing 0 spore/mL of stool (n = 1), 5 spores/mL (n = 3), 25 spores/mL (n = 3), 50 spores/mL (n = 3), 500 spores/mL (n = 2) and 5,000 spores/mL (n = 2). So, the number of DNA extractions varied with the parasite concentration tested and was higher for the lowest concentrations (Table S1). The stool aliquots were sent to the four participating laboratories at + 4 °C within 24 h of preparation.

#### DNA extraction methods

Upon reception, each center stored aliquots at + 4 °C and performed DNA extraction within two days. Each aliquot received by each laboratory was extracted. Briefly, each tube was vortexed for 15 s. Then, according to the method of each center, an adequate volume of stool was pre-treated using various methods (Table 1), and 5 μL of internal control (DiaControlDNA™, Diagenode Diagnostics) was added to the sample before DNA extraction. The elution volume was 100 µL for all methods. Main characteristics of each method are reported in Table 1. After extraction, DNA extracts were stored at + 4 °C until shipment to the coordinating center the next day.

#### Amplification

All DNA extracts were sent to the coordinating center at 4 °C where qPCR were performed the next day according to an in-house method, as previously published^22^, using a RotorGene Q (Qiagen) qPCR device. The number of PCR replicates for each DNA extract varied according to the initial concentration of spores/mL (Table S1). Each extract from specimen with 0 spore/mL were tested in duplicate by qPCR; each extract from specimen with 5, 25 or 50 spores/mL were tested six times; and each extract from specimen of 500 or 5000 spores/mL were tested eleven times. Cycle threshold (Ct) values were recorded and PCRs reactions were considered uninhibited as long as Ct value of internal control was between 24 and 30 (27 ± 3).

### Part 2

In order to optimize mechanical pretreatment, various material and diameter of beads, as well as duration and speed of bead-beating were tested.

#### Stool specimen

A positive stool for *E. bieneusi* (genotype C) was diluted with a microsporidia-negative liquid stool to obtain a range of spore concentrations of 1000, 5000 and 50,000 spores/mL.

#### Beads

Three references of beads were tested: (i) a mix containing 1.4 mm ceramic beads, 0.112 mm silica beads and a single 4 mm glass bead (MP Lysing Matrix E, MP Biomedicals); (ii) 0.1 mm zirconia and 0.5 mm garnet beads (ZR BashingBeads, Zymo Research); (iii) 0.5 mm glass beads (Next Advance). Control samples consisted in vortexed stool aliquots that were not submitted to a bead beating step before DNA extraction.

#### Bead-beating

The Tissue Lyser II (Qiagen, horizontal oscillations) was used; three oscillation frequencies were compared: 20, 25 and 30 Hz. For each frequency, three durations were applied: 60, 120 and 180 s.

#### DNA extraction

Each pretreatment protocol was performed in triplicate for each dilution (Table S1). Various conditions were tested by crossing bead beating parameters and reference of beads during three minutes. Controls consisted in samples without any pretreatment (no bead, no grinding). For mechanical pretreatment, 200 µL of stool dilution and 800 µL of lysis buffer (NucliSens® easyMAG, Biomérieux) were distributed in a tube containing beads. DNA extraction was performed with the QIAamp DNA mini kit (Qiagen) according to manufacturer’s recommendations including the use of Inhibitex buffer (Qiagen) in order to neutralize PCR inhibitors. A final elution volume of 200 µL was obtained. All DNA extracts were stored at − 80 °C till the next step.

#### Amplification

qPCR were performed in triplicate (Table S1) for each DNA extract using RotorGene Q thermocycler (Qiagen), as previously described^22^.

### Statistical analysis

The optimal number of DNA extractions and DNA amplifications for each concentration were determined according to Poisson’s law.

Statistical analyses were performed with Stata software (version 15; StataCorp, College Station, Texas, USA). All tests were two-sided, with an alpha level set at 5%. Categorical data were presented as counts and associated percentages, and continuous data as mean ± standard deviation. The percentage of positive PCRs obtained with the various protocols were compared using the Chi-squared test. When appropriate (omnibus *p*-value less than 0.05), a Marascuilo post-hoc procedure was performed. Because several PCR replicates were performed for each DNA extract, Ct values were compared using random-effects models for repeated data. Finally, Hedges’ effect sizes were calculated to illustrate the comparison of the Ct values between each type of beads and no bead beating. They were presented with their 95% confidence interval (95%CI), and interpreted according to Cohen’s recommendations: 0.2 = small effect, 0.5 = medium effect and 0.8 = large effect^23^.

### Supplementary Information

Supplementary Table S1.

Supplementary Table S2.

Supplementary Table S3.

## Data Availability

The datasets generated during and/or analysed during the current study are all presented in supplementary tables.
