# Supplementary material for: Multicenter comparative study of Enterocytozoon bieneusi DNA extraction methods from stool samples, and mechanical pretreatment protocols evaluation
Source: Sci Rep. 2024 Jul 4;14:15404. doi: 10.1038/s41598-024-66154-2 (PMC11224372; doi:10.1038/s41598-024-66154-2)
Supplement: Supplementary file 1 — Supplementary Table S1. [file 41598_2024_66154_MOESM1_ESM.docx]

**Supplementary Table S1. Design of the study**

**Part 1**

| *E. bieneusi* spores/mL of stool | Number of DNA extractions per concentration | Number of PCR per DNA extract | Number of PCR per concentration |
| --- | --- | --- | --- |
| 0 | 1 | 2 | 2 |
| 5 | 3 | 6 | 18 |
| 25 | 3 | 6 | 18 |
| 50 | 3 | 6 | 18 |
| 500 | 2 | 11 | 22 |
| 5,000 | 2 | 11 | 22 |

**Part 2**

| *E. bieneusi* spores/mL of stool | For one pair [speed/duration]* of grinding and one type of beads | | |
| --- | --- | --- | --- |
|  | Number of DNA extractions per concentrations | Number of PCR per DNA extract | Number of PCR per concentration |
| 1,000 | 3 | 3 | 9 |
| 5,000 | 3 | 3 | 9 |
| 50,000 | 3 | 3 | 9 |

*Nine pairs in total: 20 Hz/60 sec; 20 Hz/120 sec; 20 Hz/180 sec; 25 Hz/60 sec; 25 Hz/120 sec; 25 Hz/180 sec; 30 Hz/60 sec; 30 Hz/120 sec; 30 Hz/180 sec.

Sec: seconds
