# Supplementary material for: Multicenter comparative study of Enterocytozoon bieneusi DNA extraction methods from stool samples, and mechanical pretreatment protocols evaluation
Source: Sci Rep. 2024 Jul 4;14:15404. doi: 10.1038/s41598-024-66154-2 (PMC11224372; doi:10.1038/s41598-024-66154-2)
Supplement: Supplementary file 2 — Supplementary Table S2. [file 41598_2024_66154_MOESM2_ESM.docx]

**Supplementary Table S2. Ct values obtained with each method (part 1).** When PCR was negative, Ct value was indicated as « >40 ».

| **Concentration (spores/mL)** | **Eluate number (per each concentration)** | **Ct values of PCR replicates** | | | | | | |
| --- | --- | --- | --- | --- | --- | --- | --- | --- |
|  |  | **Method 1** | **Method 2** | **Method 3** | **Method 4** | **Method 5** | **Method 6** | **Method 7** |
| **0** | 1 | >40 | >40 | >40 | >40 | >40 | >40 | >40 |
|  |  | >40 | >40 | >40 | >40 | >40 | >40 | >40 |
| **5** | 1 | >40 | >40 | 32.77 | 34.13 | 34.27 | >40 | >40 |
|  |  | >40 | >40 | 34.51 | 34.47 | >40 | >40 | >40 |
|  |  | 34.75 | 33.89 | 34.82 | 33.79 | 34.01 | >40 | 34.30 |
|  |  | 35.26 | >40 | >40 | 32.60 | >40 | >40 | >40 |
|  |  | 34.35 | >40 | 34.01 | >40 | >40 | >40 | >40 |
|  |  | 35.67 | >40 | 33.07 | 32.95 | 34.41 | >40 | >40 |
|  | 2 | 35.59 | >40 | 34.64 | 31.80 | >40 | >40 | >40 |
|  |  | 35.24 | 33.82 | 33.62 | 31.81 | 34.68 | >40 | 35.17 |
|  |  | 34.48 | >40 | 34.11 | 31.92 | 34.07 | >40 | >40 |
|  |  | 35.77 | 35.67 | 33.66 | 34.29 | >40 | 36.51 | 35.79 |
|  |  | 34.65 | >40 | 33.29 | 32.08 | 33.53 | >40 | >40 |
|  |  | >40 | >40 | 33.95 | 32.54 | 34.07 | >40 | 35.60 |
|  | 3 | >40 | >40 | 35.77 | 31.24 | >40 | 34.79 | 36.07 |
|  |  | 35.32 | >40 | 35.62 | 32.94 | >40 | >40 | 35.22 |
|  |  | 34.73 | >40 | 32.60 | 33.25 | >40 | 35.12 | >40 |
|  |  | 35.60 | 39.02 | 33.94 | 34.61 | 34.59 | >40 | 35.51 |
|  |  | 35.24 | >40 | 34.10 | 35.49 | >40 | 35.70 | >40 |
|  |  | 36.12 | >40 | 32.21 | 33.15 | >40 | >40 | 36.08 |
| **25** | 1 | 34.15 | >40 | 33.68 | 30.69 | >40 | 35.57 | >40 |
|  |  | >40 | >40 | 31.43 | 32.13 | 35.03 | 34.47 | 35.94 |
|  |  | 34.82 | >40 | 32.18 | 31.82 | 36.41 | 33.16 | >40 |
|  |  | 34.02 | >40 | 33.96 | 31.68 | 34.66 | >40 | >40 |
|  |  | 35.79 | >40 | 32.82 | 33.73 | 34.50 | 37.14 | >40 |
|  |  | 35.59 | >40 | 33.15 | 32.15 | >40 | >40 | 35.55 |
|  | 2 | >40 | 34.48 | 34.21 | 32.64 | >40 | >40 | 35.89 |
|  |  | 33.57 | >40 | 32.14 | 31.31 | 33.81 | >40 | 33.72 |
|  |  | 34.24 | 39.12 | 32.29 | 34.45 | 33.27 | >40 | 33.87 |
|  |  | 34.98 | >40 | 32.32 | 31.08 | 34.68 | >40 | 34.74 |
|  |  | 35.31 | >40 | 33.00 | 32.58 | >40 | >40 | >40 |
|  |  | >40 | >40 | 33.44 | 33.51 | >40 | >40 | 35.78 |
|  | 3 | 33.92 | >40 | 31.84 | 33.01 | 34.63 | >40 | 33.89 |
|  |  | 34.14 | 39.62 | 33.86 | 34.84 | >40 | >40 | 34.25 |
|  |  | 31.70 | 33.47 | 32.41 | 32.92 | >40 | >40 | >40 |
|  |  | 34.87 | >40 | 31.71 | 33.24 | 33.97 | >40 | 35.40 |
|  |  | 34.97 | >40 | 33.73 | Not enough DNA | 34.85 | >40 | 33.73 |
|  |  | >40 | >40 | 35.57 | Not enough DNA | 32.96 | >40 | 34.58 |
| **50** | 1 | 33.39 | 39.88 | 31.74 | 30.73 | 35.23 | 32.08 | 33.32 |
|  |  | 32.32 | >40 | 30.56 | 31.04 | 32.70 | 31.88 | 33.18 |
|  |  | 32.07 | 33.66 | 32.11 | 30.51 | 34.34 | 31.62 | 33.89 |
|  |  | 32.76 | >40 | 31.58 | 29.55 | 33.11 | 32.21 | 35.15 |
|  |  | 32.51 | 34.53 | 32.08 | 31.82 | 32.59 | 32.88 | 33.47 |
|  |  | 32.28 | >40 | 31.01 | 31.93 | 33.16 | 32.17 | 35.52 |
|  | 2 | 32.38 | >40 | 32.59 | 30.16 | 34.46 | 34.45 | 32.88 |
|  |  | 32.87 | 34.52 | 31.42 | 30.70 | 31.99 | 35.63 | 33.09 |
|  |  | 31.66 | 33.27 | 32.22 | 30.20 | 32.32 | 34.58 | 32.25 |
|  |  | 35.81 | >40 | 31.55 | 31.14 | 32.51 | >40 | 32.88 |
|  |  | 34.21 | 41.15 | 31.59 | 30.40 | 33.67 | >40 | 32.53 |
|  |  | 32.75 | >40 | 31.48 | 30.24 | 33.18 | >40 | 33.74 |
|  | 3 | 33.07 | 34.21 | 30.83 | 30.53 | 33.00 | >40 | 33.39 |
|  |  | 33.40 | 34.05 | 31.35 | 30.32 | 32.65 | >40 | 33.55 |
|  |  | 33.36 | >40 | 31.77 | 30.91 | 33.61 | >40 | 33.26 |
|  |  | 32.56 | 33.62 | 31.44 | 30.46 | 32.37 | >40 | 33.56 |
|  |  | 33.07 | >40 | 31.38 | 30.07 | 33.94 | >40 | 32.60 |
|  |  | 33.14 | >40 | 31.50 | 31.18 | 34.48 | >40 | 32.56 |

| **Concentration (spores/mL)** | **Eluate number (per each concentration)** | **Ct values of PCR replicates** | | | | | | |
| --- | --- | --- | --- | --- | --- | --- | --- | --- |
|  |  | **Method 1** | **Method 2** | **Method 3** | **Method 4** | **Method 5** | **Method 6** | **Method 7** |
| **500** | 1 | 32.85 | >40 | 31.87 | 30.19 | 32.25 | 33.59 | 32.96 |
|  |  | 32.22 | >40 | 30.64 | 29.40 | 32.04 | >40 | 32.91 |
|  |  | 33.17 | >40 | 30.06 | 30.35 | 31.76 | 34.76 | 32.21 |
|  |  | 32.61 | 33.39 | 31.02 | 30.99 | 31.67 | 33.05 | 32.48 |
|  |  | 33.71 | >40 | 30.16 | 31.04 | 31.02 | 32.56 | 32.15 |
|  |  | 34.17 | >40 | 30.74 | 29.99 | 31.36 | 33.01 | 32.33 |
|  |  | 32.57 | >40 | 30.80 | 30.98 | 32.68 | 33.26 | 32.89 |
|  |  | 32.61 | 35.81 | 30.70 | 30.54 | 31.93 | 32.80 | 32.05 |
|  |  | 32.66 | >40 | 31.44 | 30.57 | 33.19 | 32.23 | 33.22 |
|  |  | 32.43 | 33.43 | 30.99 | 30.65 | 31.84 | 32.03 | 32.75 |
|  |  | 32.76 | >40 | 30.87 | 30.62 | 33.18 | 31.54 | 32.65 |
|  | 2 | 32.86 | >40 | 29.82 | 30.68 | 33.40 | 32.84 | 33.09 |
|  |  | 33.16 | >40 | 30.43 | 30.60 | 32.76 | 32.75 | 32.82 |
|  |  | 32.19 | >40 | 29.80 | 29.78 | 32.65 | 31.67 | 33.47 |
|  |  | 32.56 | >40 | 30.55 | 30.46 | 32.77 | 32.81 | 33.32 |
|  |  | 32.89 | >40 | 31.32 | 30.88 | 32.35 | 32.80 | 32.70 |
|  |  | 32.40 | >40 | 30.96 | 29.79 | 33.33 | 33.84 | 32.43 |
|  |  | 33.11 | 33.74 | 32.24 | 29.49 | 32.84 | >40 | 33.26 |
|  |  | 32.73 | >40 | 30.52 | 29.96 | 33.40 | 33.14 | 32.99 |
|  |  | 33.40 | >40 | 30.04 | 30.56 | 29.60 | 33.25 | 32.72 |
|  |  | 32.95 | >40 | 31.77 | 29.87 | 32.74 | 34.26 | 33.07 |
|  |  | 32.69 | 33.84 | 30.80 | 29.80 | 32.54 | 32.44 | 32.36 |
| **5,000** | 1 | 29.75 | 32.40 | 27.35 | 27.08 | 28.74 | 35.24 | 28.92 |
|  |  | 29.23 | 33.11 | 27.73 | 26.68 | 29.45 | 30.54 | 29.32 |
|  |  | 29.46 | 32.86 | 27.54 | 26.80 | 29.15 | 30.74 | 29.54 |
|  |  | 29.51 | 32.73 | 27.55 | 26.21 | 29.20 | 30.60 | 29.41 |
|  |  | 29.72 | 33.01 | 27.69 | 26.82 | 29.42 | 29.50 | 29.39 |
|  |  | 29.47 | 34.77 | 27.48 | 26.76 | 29.10 | 30.28 | 29.19 |
|  |  | 29.31 | 32.59 | 27.97 | 26.33 | 28.83 | 30.80 | 28.88 |
|  |  | 29.28 | 32.22 | 27.97 | 26.52 | 29.39 | 30.37 | 29.46 |
|  |  | 29.91 | 31.88 | 27.90 | 26.85 | 29.78 | 30.57 | 29.32 |
|  |  | 29.75 | 32.29 | 27.67 | 27.22 | 28.85 | 30.72 | 29.52 |
|  |  | 29.24 | 33.20 | 27.33 | 26.77 | 29.37 | 30.64 | 29.78 |
|  | 2 | 28.94 | 32.56 | 27.60 | 27.00 | 29.38 | 30.16 | 30.12 |
|  |  | 29.12 | 30.58 | 27.54 | 27.09 | 29.43 | 29.83 | 29.66 |
|  |  | 29.43 | 32.94 | 27.60 | 26.74 | 29.29 | 30.21 | 29.99 |
|  |  | 28.82 | 33.24 | 27.61 | 26.40 | 29.77 | 30.62 | 29.50 |
|  |  | 29.12 | 32.39 | 27.78 | 26.80 | 29.63 | 30.05 | 30.05 |
|  |  | 28.53 | 31.63 | 28.00 | 26.57 | 29.58 | 30.41 | 30.25 |
|  |  | 28.63 | 31.86 | 27.65 | 27.08 | 29.69 | 29.52 | 29.84 |
|  |  | 28.76 | 32.27 | 27.96 | 27.22 | 29.50 | 30.59 | 30.58 |
|  |  | 28.84 | 34.34 | 27.47 | 26.88 | 30.00 | 30.55 | 30.17 |
|  |  | 28.93 | 30.89 | 27.72 | 26.92 | 28.86 | 30.29 | 30.08 |
|  |  | 29.07 | 30.89 | 27.39 | 26.87 | 29.45 | 29.89 | 30.17 |
