# Supplementary material for: Multicenter comparative study of Enterocytozoon bieneusi DNA extraction methods from stool samples, and mechanical pretreatment protocols evaluation
Source: Sci Rep. 2024 Jul 4;14:15404. doi: 10.1038/s41598-024-66154-2 (PMC11224372; doi:10.1038/s41598-024-66154-2)
Supplement: Supplementary file 3 — Supplementary Table S3. [file 41598_2024_66154_MOESM3_ESM.docx]

**Supplementary Table S3. Ct values obtained with each mechanical pretreatment protocol (part 2).**

| **Beads** | **Spores/mL of stool** | **Grinding duration (sec)** | **Grinding speed (Hz)** | **DNA extract number** | **Ct values of replicates** | | |
| --- | --- | --- | --- | --- | --- | --- | --- |
|  |  |  |  |  | **PCR1** | **PCR2** | **PCR3** |
| Glass beads  Glass beads | 1,000 | 60 | 20 | 1 | 19.43 | 20.17 | 19.82 |
|  |  |  |  | 2 | 20.02 | 20.40 | 19.48 |
|  |  |  |  | 3 | 20.81 | 20.80 | 20.43 |
|  |  |  | 25 | 1 | 22.04 | 20.79 | 21.35 |
|  |  |  |  | 2 | 22.10 | 21.76 | 21.92 |
|  |  |  |  | 3 | 21.90 | 20.80 | 21.00 |
|  |  |  | 30 | 1 | 19.00 | 19.93 | 20.12 |
|  |  |  |  | 2 | 20.14 | 20.43 | 19.73 |
|  |  |  |  | 3 | 21.75 | 21.97 | 21.58 |
|  |  | 120 | 20 | 1 | 19.90 | 21.37 | 21.10 |
|  |  |  |  | 2 | 21.22 | 21.78 | 20.82 |
|  |  |  |  | 3 | 21.92 | 21.83 | 22.13 |
|  |  |  | 25 | 1 | 20.29 | 21.58 | 20.18 |
|  |  |  |  | 2 | 20.54 | 20.47 | 21.05 |
|  |  |  |  | 3 | 21.14 | 21.75 | 21.05 |
|  |  |  | 30 | 1 | 21.69 | 21.21 | 21.82 |
|  |  |  |  | 2 | 21.05 | 22.64 | 22.71 |
|  |  |  |  | 3 | 22.15 | 22.39 | 21.90 |
|  |  | 180 | 20 | 1 | 19.79 | 19.71 | 20.67 |
|  |  |  |  | 2 | 20.72 | 20.49 | 20.10 |
|  |  |  |  | 3 | 20.78 | 19.14 | 21.64 |
|  |  |  | 25 | 1 | 20.00 | 20.34 | 20.14 |
|  |  |  |  | 2 | 20.92 | 21.16 | 19.52 |
|  |  |  |  | 3 | 20.66 | 20.43 | 20.80 |
|  |  |  | 30 | 1 | 21.55 | 22.22 | 21.36 |
|  |  |  |  | 2 | 21.85 | 21.37 | 22.10 |
|  |  |  |  | 3 | 22.67 | 22.25 | 21.72 |
|  | 5,000 | 60 | 20 | 1 | 23.24 | 22.42 | 22.91 |
|  |  |  |  | 2 | 24.86 | 25.34 | 26.61 |
|  |  |  |  | 3 | 26.37 | 26.22 | 25.28 |
|  |  |  | 25 | 1 | 24.71 | 25.44 | 25.01 |
|  |  |  |  | 2 | 25.05 | 25.34 | 24.93 |
|  |  |  |  | 3 | 25.51 | 25.21 | 25.74 |
|  |  |  | 30 | 1 | 21.75 | 23.02 | 22.45 |
|  |  |  |  | 2 | 21.21 | 24.31 | 23.27 |
|  |  |  |  | 3 | 23.15 | 23.94 | 25.83 |
|  |  | 120 | 20 | 1 | 24.89 | 23.99 | 23.22 |
|  |  |  |  | 2 | 24.68 | 23.79 | 24.27 |
|  |  |  |  | 3 | 25.46 | 25.32 | 24.70 |
|  |  |  | 25 | 1 | 25.14 | 24.00 | 24.40 |
|  |  |  |  | 2 | 23.99 | 24.94 | 26.04 |
|  |  |  |  | 3 | 26.04 | 24.49 | 25.62 |
|  |  |  | 30 | 1 | 24.16 | 24.79 | 24.11 |
|  |  |  |  | 2 | 23.98 | 26.28 | 24.26 |
|  |  |  |  | 3 | 24.68 | 25.18 | 25.41 |
|  |  | 180 | 20 | 1 | 24.53 | 24.77 | 24.80 |
|  |  |  |  | 2 | 24.80 | 24.95 | 24.72 |
|  |  |  |  | 3 | 22.92 | 25.06 | 24.17 |
|  |  |  | 25 | 1 | 23.10 | 22.39 | 23.73 |
|  |  |  |  | 2 | 25.08 | 25.22 | 25.23 |
|  |  |  |  | 3 | 24.68 | 25.22 | 24.78 |
|  |  |  | 30 | 1 | 23.77 | 24.01 | 24.73 |
|  |  |  |  | 2 | 23.51 | 24.24 | 24.75 |
|  |  |  |  | 3 | 25.05 | 24.51 | 24.68 |
|  | 50,000 | 60 | 20 | 1 | 25.25 | 25.13 | 26.26 |
|  |  |  |  | 2 | 26.05 | 26.34 | 25.22 |
|  |  |  |  | 3 | 27.17 | 27.09 | 27.46 |
|  |  |  | 25 | 1 | 28.27 | 28.53 | 27.97 |
|  |  |  |  | 2 | 28.55 | 27.82 | 30.08 |
|  |  |  |  | 3 | 27.73 | 28.29 | 26.80 |
|  |  |  | 30 | 1 | 25.24 | 25.06 | 24.30 |
|  |  |  |  | 2 | 26.32 | 27.07 | 27.40 |
|  |  |  |  | 3 | 27.97 | 27.84 | 27.66 |
|  |  | 120 | 20 | 1 | 25.25 | 26.04 | 26.28 |
|  |  |  |  | 2 | 27.34 | 27.22 | 23.84 |
|  |  |  |  | 3 | 27.39 | 27.92 | 27.02 |
|  |  |  | 25 | 1 | 26.22 | 27.18 | 26.59 |
|  |  |  |  | 2 | 27.93 | 28.21 | 28.17 |
|  |  |  |  | 3 | 27.99 | 28.26 | 28.18 |
|  |  |  | 30 | 1 | 24.42 | 26.77 | 26.92 |
|  |  |  |  | 2 | 27.23 | 25.55 | 26.34 |
|  |  |  |  | 3 | 26.02 | 27.59 | 26.95 |
|  |  | 180 | 20 | 1 | 25.24 | 26.77 | 26.23 |
|  |  |  |  | 2 | 25.02 | 24.97 | 25.87 |
|  |  |  |  | 3 | 25.95 | 24.73 | 26.07 |
|  |  |  | 25 | 1 | 28.49 | 28.34 | 27.36 |
|  |  |  |  | 2 | 28.20 | 28.46 | 27.96 |
|  |  |  |  | 3 | 29.18 | 29.02 | 27.14 |
|  |  |  | 30 | 1 | 28.19 | 26.70 | 26.78 |
|  |  |  |  | 2 | 27.34 | 27.01 | 26.36 |
|  |  |  |  | 3 | 26.14 | 26.43 | 27.74 |

| MP Lysing Matrix E  MP Lysing Matrix E | 1,000 | 60 | 20 | 1 | 20.62 | 20.60 | 21.22 |
| --- | --- | --- | --- | --- | --- | --- | --- |
|  |  |  |  | 2 | 20.46 | 21.79 | 20.58 |
|  |  |  |  | 3 | 21.87 | 21.60 | 21.27 |
|  |  |  | 25 | 1 | 21.28 | 21.69 | 22.39 |
|  |  |  |  | 2 | 21.55 | 21.40 | 22.11 |
|  |  |  |  | 3 | 21.84 | 23.49 | 22.59 |
|  |  |  | 30 | 1 | 20.74 | 20.32 | 18.68 |
|  |  |  |  | 2 | 19.35 | 20.46 | 20.04 |
|  |  |  |  | 3 | 20.35 | 20.52 | 20.95 |
|  |  | 120 | 20 | 1 | 21.13 | 20.88 | 21.73 |
|  |  |  |  | 2 | 21.66 | 22.36 | 21.45 |
|  |  |  |  | 3 | 22.93 | 22.24 | 22.30 |
|  |  |  | 25 | 1 | 21.32 | 20.27 | 20.95 |
|  |  |  |  | 2 | 21.10 | 20.47 | 21.27 |
|  |  |  |  | 3 | 21.72 | 22.04 | 21.73 |
|  |  |  | 30 | 1 | Not enough DNA | 20.49 | Not enough DNA |
|  |  |  |  | 2 | Not enough DNA | 21.23 | Not enough DNA |
|  |  |  |  | 3 | 21.93 | 21.95 | 22.15 |
|  |  | 180 | 20 | 1 | 20.20 | 21.32 | 22.26 |
|  |  |  |  | 2 | 21.57 | 21.77 | 21.94 |
|  |  |  |  | 3 | 22.89 | 22.54 | 22.35 |
|  |  |  | 25 | 1 | 21.00 | 20.62 | 20.84 |
|  |  |  |  | 2 | 21.80 | 21.99 | 21.60 |
|  |  |  |  | 3 | 21.63 | 22.09 | 22.11 |
|  |  |  | 30 | 1 | 21.10 | 20.23 | 19.95 |
|  |  |  |  | 2 | 20.56 | 20.88 | 20.70 |
|  |  |  |  | 3 | 21.33 | 21.08 | 21.45 |
|  | 5,000 | 60 | 20 | 1 | 24.00 | 24.12 | 24.40 |
|  |  |  |  | 2 | 23.74 | 24.46 | 25.10 |
|  |  |  |  | 3 | 24.62 | 25.49 | 26.02 |
|  |  |  | 25 | 1 | 24.75 | 25.57 | 25.26 |
|  |  |  |  | 2 | 24.96 | 25.26 | 25.56 |
|  |  |  |  | 3 | 24.20 | 25.00 | 23.95 |
|  |  |  | 30 | 1 | 20.34 | 20.88 | 22.05 |
|  |  |  |  | 2 | 20.88 | 23.24 | 22.93 |
|  |  |  |  | 3 | 22.17 | 22.50 | 22.51 |
|  |  | 120 | 20 | 1 | 24.66 | 22.38 | 21.14 |
|  |  |  |  | 2 | 24.12 | 24.45 | 23.42 |
|  |  |  |  | 3 | 23.98 | 25.00 | 25.13 |
|  |  |  | 25 | 1 | 24.20 | 23.94 | 24.06 |
|  |  |  |  | 2 | 24.63 | 23.63 | 24.39 |
|  |  |  |  | 3 | 24.40 | 24.85 | 24.53 |
|  |  |  | 30 | 1 | 23.28 | 23.33 | 24.04 |
|  |  |  |  | 2 | 24.50 | 24.05 | 23.18 |
|  |  |  |  | 3 | 24.19 | 23.98 | 23.92 |
|  |  | 180 | 20 | 1 | 24.28 | 23.85 | 24.49 |
|  |  |  |  | 2 | 23.51 | 22.78 | 22.86 |
|  |  |  |  | 3 | 25.04 | 24.35 | 23.31 |
|  |  |  | 25 | 1 | 24.04 | 23.81 | 23.97 |
|  |  |  |  | 2 | 24.05 | 23.66 | 24.66 |
|  |  |  |  | 3 | 24.30 | 24.72 | 24.26 |
|  |  |  | 30 | 1 | 23.96 | 24.02 | 23.24 |
|  |  |  |  | 2 | 23.78 | 24.03 | 23.86 |
|  |  |  |  | 3 | 23.89 | 23.87 | 24.03 |
|  | 50,000 | 60 | 20 | 1 | 26.02 | 26.34 | 25.14 |
|  |  |  |  | 2 | 26.30 | 26.17 | 26.29 |
|  |  |  |  | 3 | 26.52 | 26.89 | 27.42 |
|  |  |  | 25 | 1 | 29.22 | 28.16 | 27 |
|  |  |  |  | 2 | 28.41 | 27.30 | 26.90 |
|  |  |  |  | 3 | 27.39 | 27.63 | 27.90 |
|  |  |  | 30 | 1 | 25.50 | 24.10 | 24.01 |
|  |  |  |  | 2 | 26.20 | 24.63 | 26.28 |
|  |  |  |  | 3 | 25.86 | 25.89 | 25.50 |
|  |  | 120 | 20 | 1 | 26.15 | 21.87 | 26.35 |
|  |  |  |  | 2 | 27.10 | 27.48 | 27.19 |
|  |  |  |  | 3 | 27.30 | 27.61 | 27.43 |
|  |  |  | 25 | 1 | 25.99 | 25.34 | 27.40 |
|  |  |  |  | 2 | 26.04 | 27.12 | 26.79 |
|  |  |  |  | 3 | 25.57 | 26.83 | 27.91 |
|  |  |  | 30 | 1 | 25.26 | 26.39 | 26.31 |
|  |  |  |  | 2 | Not enough DNA | 26.24 | Not enough DNA |
|  |  |  |  | 3 | 27.35 | 27.14 | 27.20 |
|  |  | 180 | 20 | 1 | 27.07 | 26.77 | 26.74 |
|  |  |  |  | 2 | 27.20 | 27.12 | 27.13 |
|  |  |  |  | 3 | 26.81 | 26.92 | 24.91 |
|  |  |  | 25 | 1 | 25.31 | 25.81 | 26.58 |
|  |  |  |  | 2 | 25.94 | 25.72 | 26.35 |
|  |  |  |  | 3 | 26.53 | 26.20 | 25.93 |
|  |  |  | 30 | 1 | 27.27 | 27.14 | 27.02 |
|  |  |  |  | 2 | 26.99 | 26.57 | 24.77 |
|  |  |  |  | 3 | 25.27 | 25.42 | 25.76 |
| ZR BashingBeads  ZR BashingBeads | 1,000 | 60 | 20 | 1 | 21.23 | 20.19 | 20.87 |
|  |  |  |  | 2 | 20.69 | 21.41 | 21.04 |
|  |  |  |  | 3 | 20.99 | 21.28 | 21.09 |
|  |  |  | 25 | 1 | 21.22 | 21.24 | 21.73 |
|  |  |  |  | 2 | 21.73 | 22.40 | 21.84 |
|  |  |  |  | 3 | 21.22 | 21.10 | 21.17 |
|  |  |  | 30 | 1 | 18.28 | 19.23 | 19.04 |
|  |  |  |  | 2 | 19.54 | 19.88 | 18.56 |
|  |  |  |  | 3 | 20.89 | 19.03 | 18.84 |
|  |  | 120 | 20 | 1 | 21.09 | 20.62 | 20.77 |
|  |  |  |  | 2 | 20.76 | 21.54 | 21.47 |
|  |  |  |  | 3 | 21.02 | 21.10 | 22.79 |
|  |  |  | 25 | 1 | 20.69 | 20.59 | 20.01 |
|  |  |  |  | 2 | 20.45 | 21.34 | 21.09 |
|  |  |  |  | 3 | 20.83 | 21.43 | 21.08 |
|  |  |  | 30 | 1 | 20.09 | 19.13 | 19.99 |
|  |  |  |  | 2 | 20.83 | 20.99 | 20.40 |
|  |  |  |  | 3 | 21.63 | 20.37 | 21.11 |
|  |  | 180 | 20 | 1 | 21.99 | 21.17 | 20.69 |
|  |  |  |  | 2 | 20.59 | 20.85 | 19.75 |
|  |  |  |  | 3 | 20.26 | 21.84 | 21.53 |
|  |  |  | 25 | 1 | 18.86 | 20.77 | 20.30 |
|  |  |  |  | 2 | 20.06 | 20.78 | 21.32 |
|  |  |  |  | 3 | 20.62 | 20.96 | 21.01 |
|  |  |  | 30 | 1 | 20.02 | 20.67 | 21.06 |
|  |  |  |  | 2 | 21.08 | 20.12 | 21.44 |
|  |  |  |  | 3 | 21.65 | 20.66 | 21.82 |
|  | 5,000 | 60 | 20 | 1 | 23.42 | 22.76 | 23.55 |
|  |  |  |  | 2 | 23.22 | 23.95 | 23.94 |
|  |  |  |  | 3 | 23.69 | 23.81 | 24.80 |
|  |  |  | 25 | 1 | 24.44 | 24.65 | 25.58 |
|  |  |  |  | 2 | 25.65 | 25.18 | 25.69 |
|  |  |  |  | 3 | 24.21 | 25.12 | 24.72 |
|  |  |  | 30 | 1 | 23.12 | 21.76 | 22.72 |
|  |  |  |  | 2 | 22.86 | 22.65 | 22.79 |
|  |  |  |  | 3 | 23.03 | 22.57 | 23.29 |
|  |  | 120 | 20 | 1 | 24.49 | 22.92 | 23.86 |
|  |  |  |  | 2 | 25.01 | 25.34 | 25.40 |
|  |  |  |  | 3 | 26.14 | 24.74 | 25.43 |
|  |  |  | 25 | 1 | 24.44 | 23.93 | 24.09 |
|  |  |  |  | 2 | 24.75 | 24.59 | 23.99 |
|  |  |  |  | 3 | 24.51 | 24.31 | 24.66 |
|  |  |  | 30 | 1 | 22.84 | 23.18 | 23.77 |
|  |  |  |  | 2 | 24.60 | 22.10 | 23.20 |
|  |  |  |  | 3 | 23.10 | 23.77 | 23.65 |
|  |  | 180 | 20 | 1 | 23.51 | 23.96 | 24.55 |
|  |  |  |  | 2 | 23.97 | 23.59 | 23.97 |
|  |  |  |  | 3 | 23.81 | 23.74 | 23.99 |
|  |  |  | 25 | 1 | 23.20 | 24.54 | 24.06 |
|  |  |  |  | 2 | 24.38 | 24.25 | 23.79 |
|  |  |  |  | 3 | 25.22 | 24.37 | 25.16 |
|  |  |  | 30 | 1 | 22.43 | 23.17 | 24.03 |
|  |  |  |  | 2 | 23.74 | 23.92 | 24.54 |
|  |  |  |  | 3 | 24.29 | 25.23 | 24.79 |
|  | 50,000 | 60 | 20 | 1 | 26.25 | 26.66 | 26.52 |
|  |  |  |  | 2 | 26.17 | 26.99 | 26.63 |
|  |  |  |  | 3 | 26.11 | 27.33 | 26.82 |
|  |  |  | 25 | 1 | 28.60 | 28.29 | 27.49 |
|  |  |  |  | 2 | 27.56 | 27.79 | 25.90 |
|  |  |  |  | 3 | 27.36 | 27.33 | 27.36 |
|  |  |  | 30 | 1 | 25.12 | 25.42 | 25.32 |
|  |  |  |  | 2 | 25.05 | 25.01 | 25.84 |
|  |  |  |  | 3 | 24.64 | 25.56 | 26.55 |
|  |  | 120 | 20 | 1 | 26.07 | 25.79 | 26.03 |
|  |  |  |  | 2 | 26.05 | 26.68 | 25.91 |
|  |  |  |  | 3 | 27.57 | 27.32 | 27.79 |
|  |  |  | 25 | 1 | 27.35 | 25.70 | 26.45 |
|  |  |  |  | 2 | 26.62 | 25.80 | 27.85 |
|  |  |  |  | 3 | 27.50 | 27.08 | 27.22 |
|  |  |  | 30 | 1 | 27.32 | 26.17 | 25.26 |
|  |  |  |  | 2 | 26.00 | 26.81 | 27.08 |
|  |  |  |  | 3 | 26.42 | 26.91 | 26.68 |
|  |  | 180 | 20 | 1 | 27.12 | 27.28 | 26.94 |
|  |  |  |  | 2 | 27.57 | 27.32 | 27.14 |
|  |  |  |  | 3 | 26.49 | 23.95 | 25.43 |
|  |  |  | 25 | 1 | 25.77 | 25.75 | 27.13 |
|  |  |  |  | 2 | 27.42 | 26.51 | 25.51 |
|  |  |  |  | 3 | 27.81 | 27.25 | 26.37 |
|  |  |  | 30 | 1 | 28.13 | 26.99 | 26.63 |
|  |  |  |  | 2 | 27.23 | 26.46 | 27.67 |
|  |  |  |  | 3 | 26.65 | 26.90 | 25.78 |

| none (controls) | 1,000 | NA | NA | 1 | 22.01 | 20.91 | 21.51 |
| --- | --- | --- | --- | --- | --- | --- | --- |
|  |  |  |  | 2 | 21.59 | 20.89 | 21.97 |
|  |  |  |  | 3 | 20.82 | 21.92 | 21.93 |
|  | 5,000 |  |  | 1 | 25.14 | 24.39 | 25.90 |
|  |  |  |  | 2 | 25.85 | 26.54 | 26.55 |
|  |  |  |  | 3 | 27.31 | 25.49 | 27.42 |
|  | 50,000 |  |  | 1 | 26.83 | 28.77 | 26.35 |
|  |  |  |  | 2 | 26.65 | 27.95 | 27.56 |
|  |  |  |  | 3 | 27.70 | 28.58 | 28.35 |

NA : not applicable
